# Heat shock proteins at the crossroads of endosomal trafficking pathways

**DOI:** 10.1007/s10565-025-10101-y

**Published:** 2025-12-11

**Authors:** Francesca Zuppini, Lucia Renzullo, Francesca Tornatore, Pietro Poggio, Mara Brancaccio

**Affiliations:** Department of Molecular Biotechnology and Health Sciences, University of Turin, Turin, Italy

**Keywords:** Endocytosis, Heat shock proteins, Chaperones, Vesicular trafficking, Protein trafficking, HSP90, Rab

## Abstract

Cells respond to a variety of environmental stressors, including oxidative stress, nutrient deprivation, hypoxia and pathogenic invasion, which challenge cellular homeostasis and trigger adaptive responses. One of the first and most conserved effects is the activation of the heat shock response, which induces the transcription of heat shock proteins (HSPs), molecular chaperones involved in protein folding, assembly and turnover. Beyond their canonical role in maintaining proteostasis, HSPs also exert housekeeping functions, including endocytosis, a process essential for molecule internalization, nutrient uptake, receptor recycling, membrane turnover and cell migration. In this review, we explore the emerging roles of chaperone proteins in endocytic trafficking, with a particular focus on HSP90, HSP70 and small HSPs. We also highlight open questions like their attitude to act in cooperation or competition, and their propensity to form dynamics complexes. In addition, we discuss evidence suggesting that the involvement of these chaperones renders the endocytic process sensitive to stress, speculating on the role of HSPs in endocytosis as an integral component of the cellular stress response. Although some of the molecular mechanisms are still unclear, the available data reveal promising and interesting directions for further research.

**Graphical Abstract:**

Graphical headlights

• Heat shock proteins (HSPs) regulate multiple steps of endocytosis, including cargo internalization, recycling, translocation across endosomal membrane and stabilization of core components of endocytic machinery

• Some HSPs can act either cooperatively or antagonistically

• HSP-membrane interactions and lipid specificity may drive functional specialization in vesicle trafficking

• Stress reprograms HSP activity, making endocytosis a stress-regulated process that supports cell survival

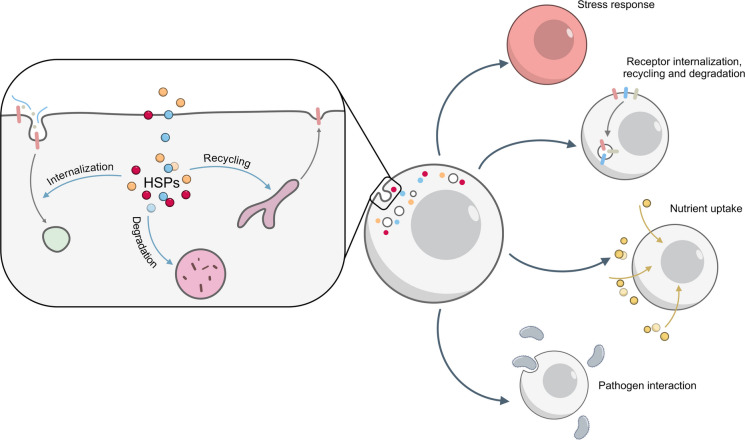

## Introduction

### Endocytosis

#### Mechanisms of endocytic uptake

**Fig. 1:**
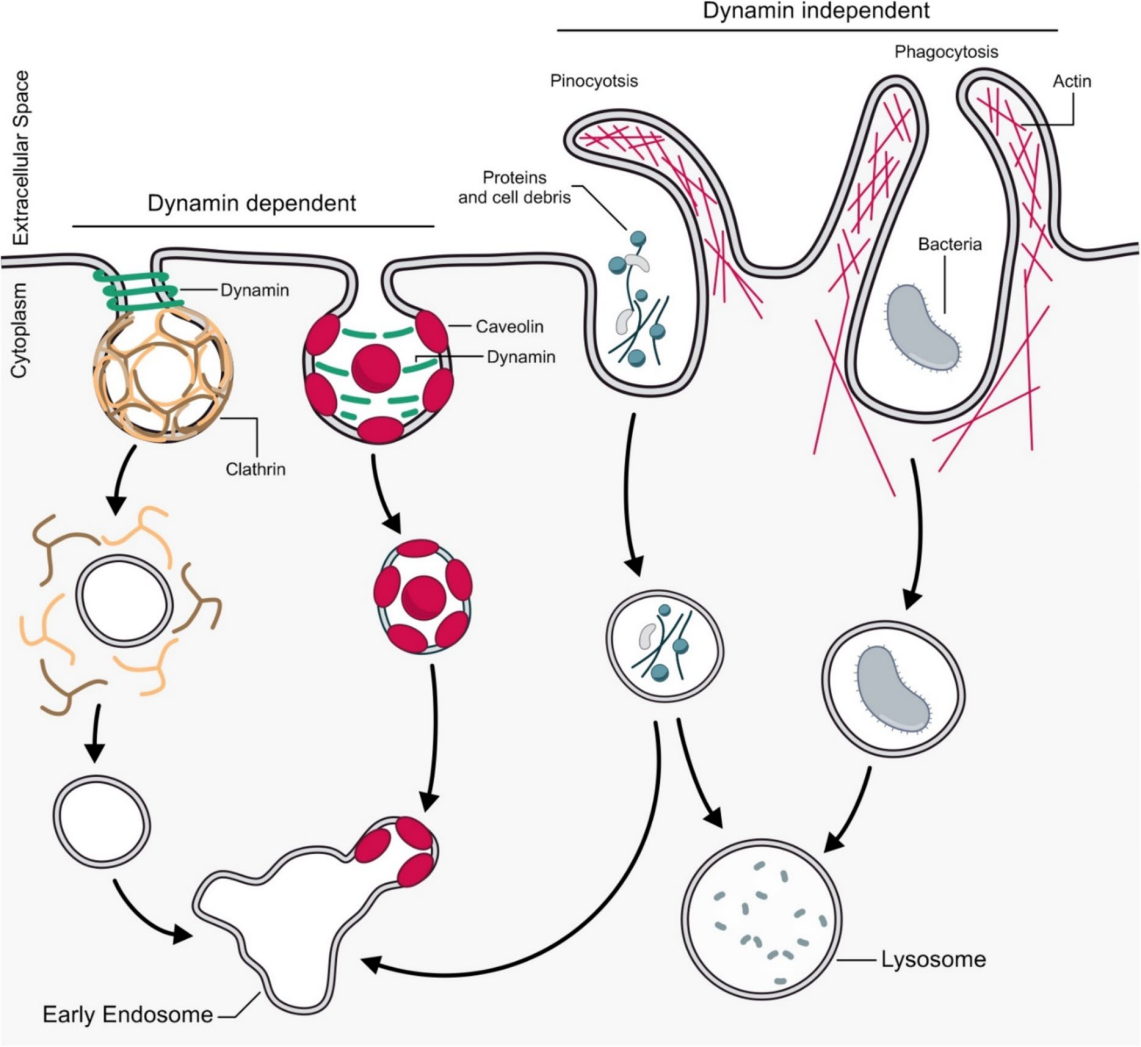
Mechanisms of endocytosis. Endocytic pathways are classified, based on the involvement of dynamin, into dynamin-dependent and dynamin-independent mechanisms. Dynamin is a multidomain GTPase recruited to coated pits where it mediates the formation, fission and release of vesicles. Dynamin-dependent endocytosis typically involves clathrin- or caveolin-mediated mechanisms, where both clathrin and caveolin surround the vesicle and mediate plasma membrane (PM) deformation. In clathrin-mediated endocytosis (CME), clathrin is recruited to cytosolic receptor domains, initiating the formation of a clathrin-coated pit. Then dynamin becomes enriched at the neck of the pit and mediates fission. On the other hand, formation of caveolae is dependent on caveolin proteins which oligomerize on the membrane and create the pit. In caveolae, dynamin is associated with the bulb and does not become enriched at the neck. After internalization, these pathways merge into early endosomes before undergoing sorting, where material may be sent back to the surface or onwards to the late endosome and lysosome. Dynamin-independent pathways rely on other factors and include processes like pinocytosis and phagocytosis. Pinocytosis is generally a stimulated pathway involving uptake of large volumes of fluid. Phagocytosis occurs after a binding event at the cell surface and is characterized by engulfment of large particles. Both these processes trigger actin polymerization to form a vesicle around the bound material. Phagosomes fuse directly with lysosomes, while pinosomes can either fuse directly with lysosomes or follow the early endosome route

Endocytosis is a vital cellular process that governs the uptake and regulation of extracellular molecules, surface proteins and receptors, impacting numerous physiological functions such as adhesion, migration, antigen presentation and cell signaling (Mukherjee et al. 1997; O’Sullivan and Lindsay 2020). The different endocytic pathways have been classified based either on the morphological features of the membranous structures involved or on their requirement for certain key components (Basturea 2019). Classically, endocytic pathways have been defined by their dependence on dynamin, enabling categorization into dynamin-dependent and dynamin-independent. Dynamin is a GTPase protein that, during membrane invagination and vesicle formation, binds to and assembles around the neck of the endocytic vesicle forming a polymer. GTP binding to dynamin and subsequent hydrolysis cause a conformational change in the dynamin polymer that thus constricts the vesicle neck resulting in membrane scission (Basturea 2019). Dynamin dependent endocytosis may rely on the presence of the coat protein clathrin and is therefore categorized as clathrin mediated endocytosis (CME) and clathrin independent endocytosis (CIE). Clathrin is composed of three heavy chains and three light chains arranged in a triskelion shape; when the triskelia interact, they form a polyhedral lattice that promotes membrane deformation and surrounds the vesicle (Defelipe et al. 2024). Alternatively, dynamin-dependent endocytosis may be independent of clathrin but could rely on the presence of proteins that belong to the caveolin protein family. Caveolins are a family of integral membrane proteins that mediate the formation of plasma membrane (PM) invaginations, called caveolae. Oligomers of caveolin form the coat of these invaginations, mediating membrane deformation and curvature to form caveolin-coated vesicles (Thomas and Smart 2008). It has been observed that dynamin is not associated with the neck of caveolin-coated vesicles, but it is enriched at the bulb; in this case dynamin does not mediate the fission of the vesicle but it stabilizes the caveolae through a mechanism that is independent of GTP hydrolysis (Parton et al. 2024) (Fig. 1).

Several endocytic pathways are dynamin independent and rely on the activity of different proteins, including actin, small GTPases such as ADP-ribosylation factor 6 (ARF6) and Cell division cycle 42 (CDC42), the membrane-associated flotillins and secondary messengers such as calcium (Gundu et al. 2022). Pinocytosis, also known as fluid phase endocytosis, is a dynamin-independent mechanism of endocytosis, a form of cellular “drinking” where the cell mediates the non-selective uptake of solute molecules or nutrients internalizing extracellular fluid. Pinocytosis is further classified into micropinocytosis and macropinocytosis, based on the diameter of the vesicles and the amount of solute uptake (Gundu et al. 2022). Larger particles are internalized through a different dynamin-independent mechanism known as phagocytosis; this process is mainly used by cells to engulf pathogens and cellular debris (Egami 2016).

### Intracellular endosomal sorting

Once internalized, cargoes are sorted within the cell, primarily at the early endosomes (EEs), also known as sorting endosomes (SEs), which serve as central hubs from which different trafficking routes diverge. These organelles are characterized by their dynamic and rapid fusion capabilities, essential for efficient cargo transport (Murphy et al. 1984). From EEs, cargoes can follow three main intracellular trafficking pathways: recycling back to the plasma membrane, degradation via lysosomes or retrograde transport to the Golgi apparatus. These sorting decisions are tightly regulated, ensuring proper cellular homeostasis.

In the recycling pathway cargo proteins can return to the plasma membrane through either a fast- or slow- recycling route. The fast-recycling pathway directly transports back proteins and molecules from EEs to the plasma membrane (Murphy et al. 1984). In this context, a broad role is played by the retromer complex (Follett et al. 2017), a heteropentamer composed of a Vacuolar protein sorting (VPS) trimer (consisting of VPS26, VPS29, VPS35) and a sorting nexin (SNX) dimer. VPS proteins bind to transmembrane endosomal proteins that have to be transported, while SNX proteins stabilize the association of the retromer complex to the endosomal membrane (Vagnozzi and Praticò, 2019; Worby and Dixon 2002). Consequently, the complex facilitates the return of endosomal transmembrane proteins to the cell surface.

The slow-recycling pathway, instead, occurs via the formation of recycling endosomes (REs) from EEs. In most cells, the EEs elongate and assume a tubular shape, giving rise to REs. These organelles usually organize into a complex structure which localizes in the perinuclear area, named endocytic recycling compartment (ERC), from which cargo-containing vesicles originate and are trafficked back to the PM (Hsu and Prekeris 2010).

In both recycling pathways, the presence of sorting motifs directs them to recycling. Sorting motifs are short, conserved amino acid sequences, located in the cytoplasmic tail of cargoes, that function as signals for intracellular trafficking. Common sorting motifs include tyrosine-based motifs, dileucine motifs and ubiquitin tags. These signals are recognized by specific adaptor proteins and sorting complexes, which facilitate the inclusion of cargo into vesicles physically connecting the cargo to the protein coat that assembles on the budding membrane. This ensures that the cargo is incorporate into vesicles that are targeted to specific intracellular compartments (Weeratunga et al. 2020; Cullen and Steinberg 2018).

In the degradative pathway, vesicles containing cargoes destined for degradation undergo a maturation process, evolving from EEs into late endosomes (LEs). During this transition, EEs acquire distinct molecular markers and experience changes in membrane composition, pH and morphology. Initially, EEs are characterized by a tubular shape and neutral pH, but as they mature, they become more spherical and acidic, hallmarks of LE formation (Frankel and Audhya 2018; Kobayashi et al. 1998). As LEs mature, they generate intraluminal vesicles (ILVs) by inward budding of the endosomal membrane, evolving into multivesicular bodies (MVBs), that fuse with lysosomes (Frankel and Audhya 2018; Kobayashi et al. 1998).

**Fig. 2:**
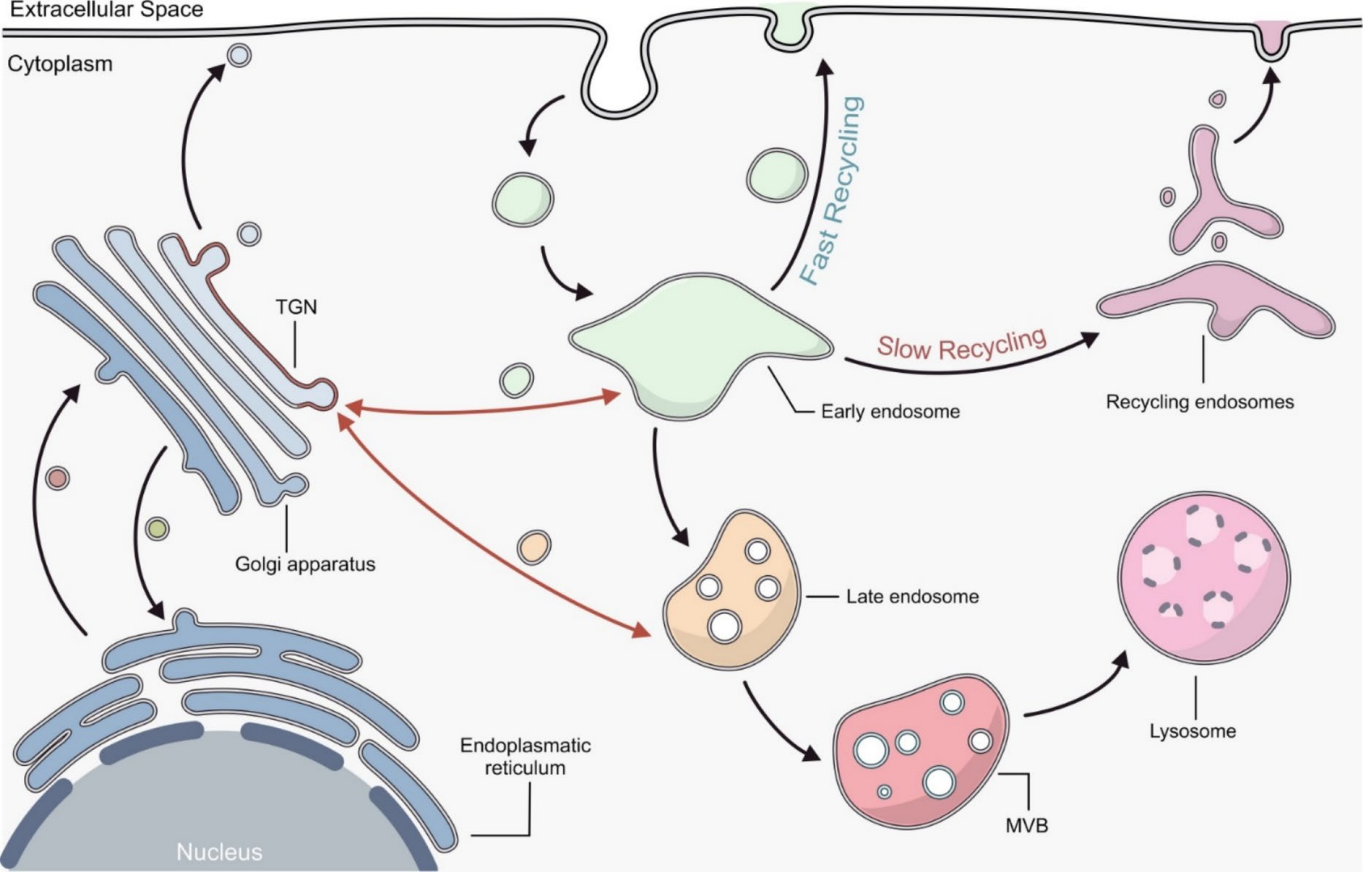
Endocytic pathways. Once internalized, cargoes enter early endosomes (EEs) where most sorting events are initiated. Selected cargo can be recycled back to the cell surface either directly, in a process termed “fast recycling,” or by transit through the endocytic recycling compartment, termed “slow recycling.” Other cargoes are targeted for degradation within the lysosome. EEs undergo maturation through the formation of intraluminal vesicles (ILVs), giving rise to late endosomes (LEs). LEs further mature into multivesicular bodies (MVBs) that can fuse with lysosomes to form endolysosomes within which ILVs and their associated cargoes are degraded. Additionally, cargoes from EEs and LEs may be rerouted to the Golgi apparatus via the trans-Golgi network (TGN), from where cargoes can return to the cell surface

Finally, in the retrograde transport, cargoes are directed toward the trans-Golgi network (TGN), the terminal Golgi compartment, composed by a network of tubulo-vesicular membranes (Ford et al. 2021). The TGN represents a connection hub between protein biosynthesis and endocytic transport: it receives both newly synthetized proteins and lipids from the endoplasmic reticulum, as well as molecules from the EEs. Cargoes reaching the TGN from the EEs can either be sorted to the PM by fusion with REs or degraded inside LEs and lysosomes (Shimizu et al. 2021; Gu et al. 2001) (Fig. 2).

### Rab proteins as endocytic regulators

**Fig. 3:**
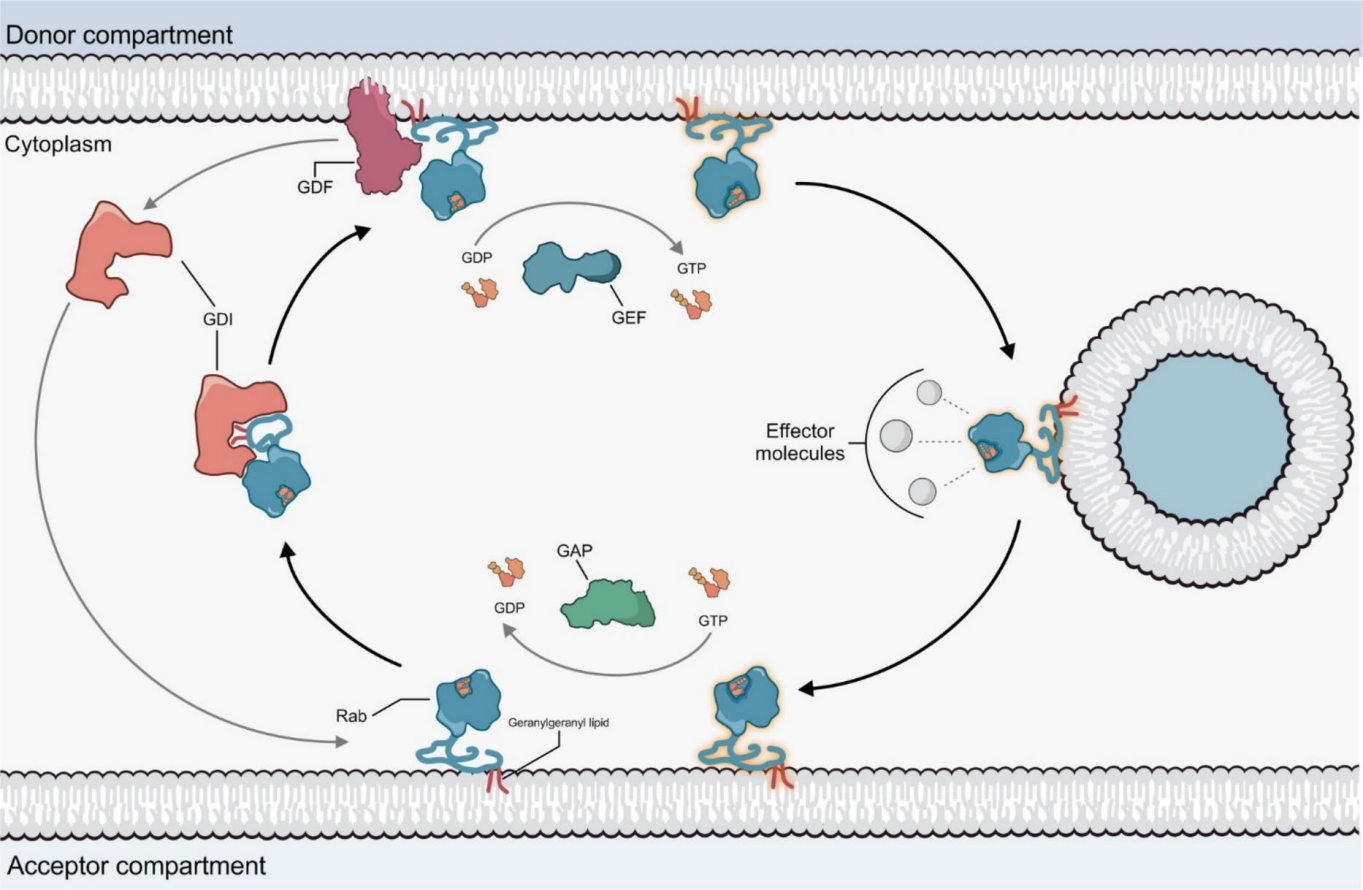
Rab protein recycling between membrane compartments. Rab proteins cycle between an inactive GDP-bound state and an active GTP-bound state and are anchored in membranes by a geranylgeranyl lipid. In their inactive form, the Rab is extracted from the acceptor membrane by GDI, which transports the Rab through the cytosol and reinserts it in the donor membrane to complete the cycle. Targeting of the Rab–GDI complex to specific membranes is mediated by interaction with a membrane-bound GDI displacement factor (GDF) (Stenmark 2009). After GDI displacement, Rab is recruited to the donor membrane where it is activated through the action of a GEF that promotes the exchange between GDP and GTP. Active GTP-bound Rab is then recruited by a budding vesicle, severed from the donor membrane, and is now able to recruit intracellular effectors. The vesicle fuses with the appropriate acceptor membrane where the Rab is inactivated through the action of GAPs, which stimulate GTP hydrolysis, turning Rab back to its GDP-bound state

Key regulators of most endocytic steps are Rab proteins, members of the Ras superfamily of small GTPases. They control vesicle formation, fusion and trafficking by cycling between an active GTP-bound state and an inactive GDP-bound state. Each Rab protein is localized to the cytoplasmic surface of a distinct membrane organelle and regulates a specific transport pathway (Maxfield and McGraw 2004). They are anchored to the membrane through a geranylgeranyl (GG) lipid group covalently linked to an amino acid (Chen and Balch 2006). Rab activity is controlled by three classes of regulators: the GTPase activating proteins (GAPs) that stimulate Rab GTPase activity, the guanine nucleotide exchange factors (GEFs) that promote the exchange of GDP to GTP and the guanine nucleotide dissociation inhibitor (GDIs) that regulate the binding and the unloading of the Rab proteins from the membrane (Yuan and Song 2020). In mammals there are three different isoforms of GDI: βGDI (ubiquitously expressed), δGDI (found in muscle and adipocyte cells) and αGDI (enriched in brain) (Shisheva et al. 1994). The GDIs bind to the GDP-bound form of Rab, causing its detachment from the membrane (Fig. 3).

Besides the well-known network of proteins that regulate endocytosis, there are other molecules, including the family of Heat shock proteins (HSPs), that play relevant functions in this process. Molecular chaperones have emerged as key elements in endocytic trafficking, not only maintaining the folding and stability of cargo proteins but also assisting their translocation across endosomal membranes. In addition, chaperones contribute to the assembly, stabilization, and recycling of core components of the endocytic machinery, thereby ensuring the efficiency of intracellular trafficking.

### Heat shock proteins

Heat shock proteins (HSPs) are components of the protein quality control system, a widespread mechanism that controls several processes such as protein synthesis, folding, unfolding, degradation and turnover. By assisting proper protein folding, preventing aggregation of misfolded proteins and disassembling aberrant protein aggregates, HSPs maintain proteostasis and overall cellular homeostasis (Hartl and Hayer-Hartl 2009). The expression of most chaperones is constitutive in cells, but it can be also induced by various stress conditions such as hypoxia, acidosis, heavy metal and oxidative damage through the activation of the heat shock response (HSR). This process is primarily mediated heat shock factor 1 (HSF1), a key transcription factos that acts as cell stress sensor (Gomez-Pastor et al. 2018). Under normal conditions, HSF1 is maintained in an inactive state in the cytosol bound to HSPs (Alasady and Mendillo 2024). Upon stress, HSF1 is released from the HSP-HSF complexes and is free to oligomerize, translocate to the nucleus and bind to the heat shock elements (HSE) on HSP promoter regions triggering their rapid transcription. However, not all the HSPs are regulated by HSF1 at the transcription level; posttranscription and translational regulations are also key mechanisms for HSP expression (Kmiecik and Mayer 2022; Hästbacka et al. 2025; De Maio 1999).

Nowadays, HSPs have been classified according to their molecular weight in small and large heat shock proteins and their specific names are given by adding to the acronym HSP a number representing their molecular weight. Small heat shock proteins (sHSPs) have a molecular weight ranging from 15 to 30 kDa, while large HSPs comprise proteins heavier than 30–40 kDa, belonging to the HSP40, HSP60, HSP70, HSP90 and HSP110 families (Huang et al. 2008). sHSPs constitute a family of ten members, named sequentially from HSPB1 to HSPB10. The HSP40 (DNAJ) family is the largest in humans and is divided into three subfamilies based on domain composition: four type A proteins, 14 type B proteins and 22 type C DNAJ members (Kampinga et al. 2009). The HSP60 family contains two major groups of chaperonins: Group I chaperonin (HSPD1) and Group II chaperonins (Kampinga et al. 2009). The HSP70 (HSPA) family comprises 13 members with different subcellular localizations and functions (Brocchieri et al. 2008). The HSP90 (HSPC) family includes five members (Kampinga et al. 2009) while the HSP110 (HSPH) family consists of four proteins with high homology to HSPA differing mainly by the presence of a long linker between the N-terminal and the C-terminal domains (Kampinga et al. 2009) (Table 1).

**Table 1:** HSP family composition, cellular localization and function

Family	Members	Subcellular localization	Function
Small HSPs	HSPB1-HSPB10	Cytosol Mitochondria Nucleus	ATP-independent sequestration of misfolded proteins to prevent aggregation
HSP40/DNAJ	DNAJA DNAJB DNAJC	Cytosol Mitochondria Nucleus	Substrate recruitment to HSP70 and HSP70 ATPase activity regulation
HSP60	Chaperonin I Chaperonin II	Mitochondria, chloroplast Cytosol	ATP-dependent folding and prevention of aggregation
HSP70	HSPA1A/1B HSPA1L HSPA2 HSPA6 HSPA7 HSPA8 HSPA12A/12B HSPA13 HSPA14 HSPA5 HSPA9	Cytosol Nucleus ER Mitochondria	ATP-dependent folding, prevention of aggregation and intermediate stabilization
HSP90	HSP90A HSP90B GRP94 TRAP1	Cytosol/Nucleus Cytosol/Nucleus Cytosol/ER Mitochondria	ATP-dependent folding of de novo synthesized proteins and refolding of misfolded proteins
HSP110	HSPH1 HSPH2 HSPH3 HSPH4	Cytosol Cytosol Cytosol ER	ATP-dependent prevention of aggregation; co-chaperone of HSP70

HSPs can also be divided into ATP-dependent and ATP-independent based on their requirement for ATP to assist polypeptide folding. sHSPs function as “holdases” promoting the stabilization of client proteins and preventing their aggregation in an ATP-independent manner (de Graff et al. 2020; Hartl et al. 2011). Among the large HSPs, the HSP40 family functions without ATP, mainly by recruiting substrates to HSP70 and acting as its co-chaperone. All the other HSP families, including HSP60, HSP70, HSP90 and HSP110, require ATP binding and hydrolysis to act on substrates, also called client proteins (Mayer 2010; Whitesell and Lindquist 2005).

HSPs not only ensure proteostasis, but also participate in several cellular events, among them signal transduction, DNA repair, protein translation, protein sorting, and trafficking (Hu et al. 2022). In this review, we aim to highlight the intersection between HSPs and endosomal trafficking pathways, with particular emphasis on three major families: HSP90, HSP70, and the sHSPs.

### HSP90

#### Isoforms, structure and client interactions

HSP90 exists as both constitutive (HSP90β) and stress-inducible (HSP90α) isoforms, whose relative expression varies depending on cell type and stress conditions (Schopf et al. 2017). These isoforms are primarily localized in the cytoplasm and nucleus, but can also be secreted into the extracellular environment, collectively referred to as extracellular HSP90 (eHSP90). eHSP90 may exist in a soluble form, associate with phospholipids at the plasma membrane, or localize to the surface and lumen of exosomes (Poggio et al. 2021; Shevtsov et al. 2020; Tang et al. 2019). In addition to HSP90α and HSP90β, multicellular organisms also express compartment-specific paralogs: glucose-regulated protein 94 (GRP94) in the endoplasmic reticulum, TNF receptor-associated protein 1 (TRAP1) in mitochondria (Sager et al. 2022), and HSP90C in the chloroplasts of plants (Mayer and Le Breton 2015). Despite their diverse localization, all these isoforms share a high degree of sequence identity and remarkable evolutionary conservation (Johnson 2012).

**Fig. 4:**
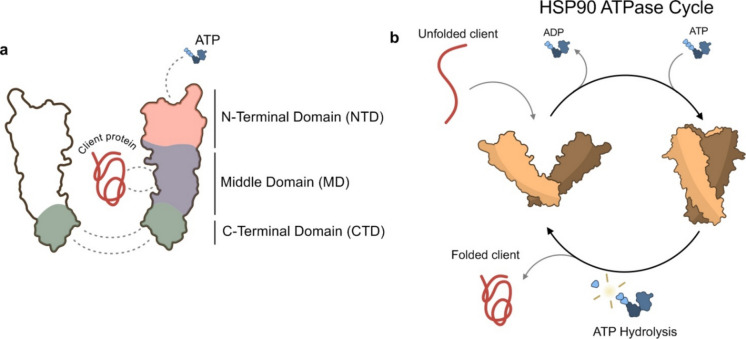
Structure, domains and ATPase cycle of HSP90. (**a**) HSP90 is composed of 3 main domains: an N-terminal domain (NTD), able to bind and hydrolyse ATP, a middle domain (MD) that provides docking sites for client proteins and a carboxy-terminal domain (CTD) that contains the dimerization motif. (**b**) HSP90 ensures that client proteins fold and assemble correctly. In the open conformation, HSP90 binds the unfolded client and ATP. ATP binding to the open state promotes NTD–NTD association (closed conformation), generating an ATPase-competent HSP90 dimer. Upon ATP hydrolysis, ADP remains bound to the NTD of each monomer, leading to dissociation of the two NTDs and regeneration of the open conformation, releasing the properly folded client

HSP90 functions as a homodimer and the dimerization is essential for its chaperoning activity in vivo (Wayne and Bolon 2007). The monomeric form of HSP90 consists of three different domains: a highly conserved N-terminal domain (NTD), a middle domain (MD) and a carboxy-terminal domain (CTD). The NTD contains the ATPase domain able to bind to ATP and promote its hydrolysis. A portion of the NTD forms a “lid” that closes the nucleotide binding pocket when bound to ATP. Efficient ATP hydrolysis also requires specific residues in the MD to keep contact with the nucleotide binding pocket. NTD and MD domains are connected through a disordered region, named charged linker (CL). The CTD is necessary for dimerization and binding to co-chaperones (Picard 2002; Schopf et al. 2017; Chiosis et al. 2023) (Fig. 4a).

ATP hydrolysis drives HSP90 conformational changes during its cycle. The open V-shape, with high rotational freedom between NTD, MD and CTD, allows ATP binding, leading to closure of the “lid” and a compact, twisted conformation where the NTDs interact (Mayer and Le Breton 2015; Taipale et al. 2010). ATP hydrolysis results in an intermediate open state and ADP dissociation restores the original open form (Fig. 4b).

HSP90 activity is also regulated by post-translational modifications like phosphorylation, acetylation and nitrosylation, which can either facilitate or inhibit client maturation. Over 20 co-chaperones modulate HSP90 function by stimulating ATPase hydrolysis, coordinating interactions with other chaperones and recruiting client proteins (Mollapour and Neckers 2012; Backe et al. 2020).

HSP90 interacts with a wide range of client proteins, including protein kinases (such as SRC, EGFR, BRAF and ERBB2) (Brugge et al. 1981; Taipale et al. 2010) and nuclear steroid receptors (like glucocorticoid, progesterone, estrogen and androgen receptors) (Schuh et al. 1985; Joab et al. 1984; Sanchez et al. 1985). It also associates with endothelial nitric oxide synthase (eNOS), telomerase reverse transcriptase (TERT), transcription factors such as p53, signal transducer and activator of transcription 3 (STAT3) and chromatin proteins (García-Cardeña et al. 1998; Holt et al. 1999; Minet et al. 1999; Sato et al. 2003; Sepehrnia et al. 1996; Tariq et al. 2009). In plants, HSP90, along with the co-chaperone suppressor of G2 allele of Skp1 (SGT1), stabilizes NOD-like receptors (NLR), proteins crucial for innate immunity. Similarly, mammalian NLRs interact with HSP90 and SGT1 that are essential for receptor signalling activity (Lu et al. 2003; Mayor et al. 2007; Takahashi et al. 2003).

Role in endocytic vesicular trafficking.

Here we will focus on another HSP90 function that is the regulation of endocytic vesicular trafficking. Although large-scale studies directly linking HSP90 to endocytic networks are currently lacking, it is well established that HSP90 interacts with multiple proteins involved in vesicle formation, cargo sorting, and endosomal transport, including SNX family members, actin regulators and VPS proteins (Mankovich and Freeman 2022).

### Regulation of Rab GTPases and receptor uptake

**Fig. 5:**
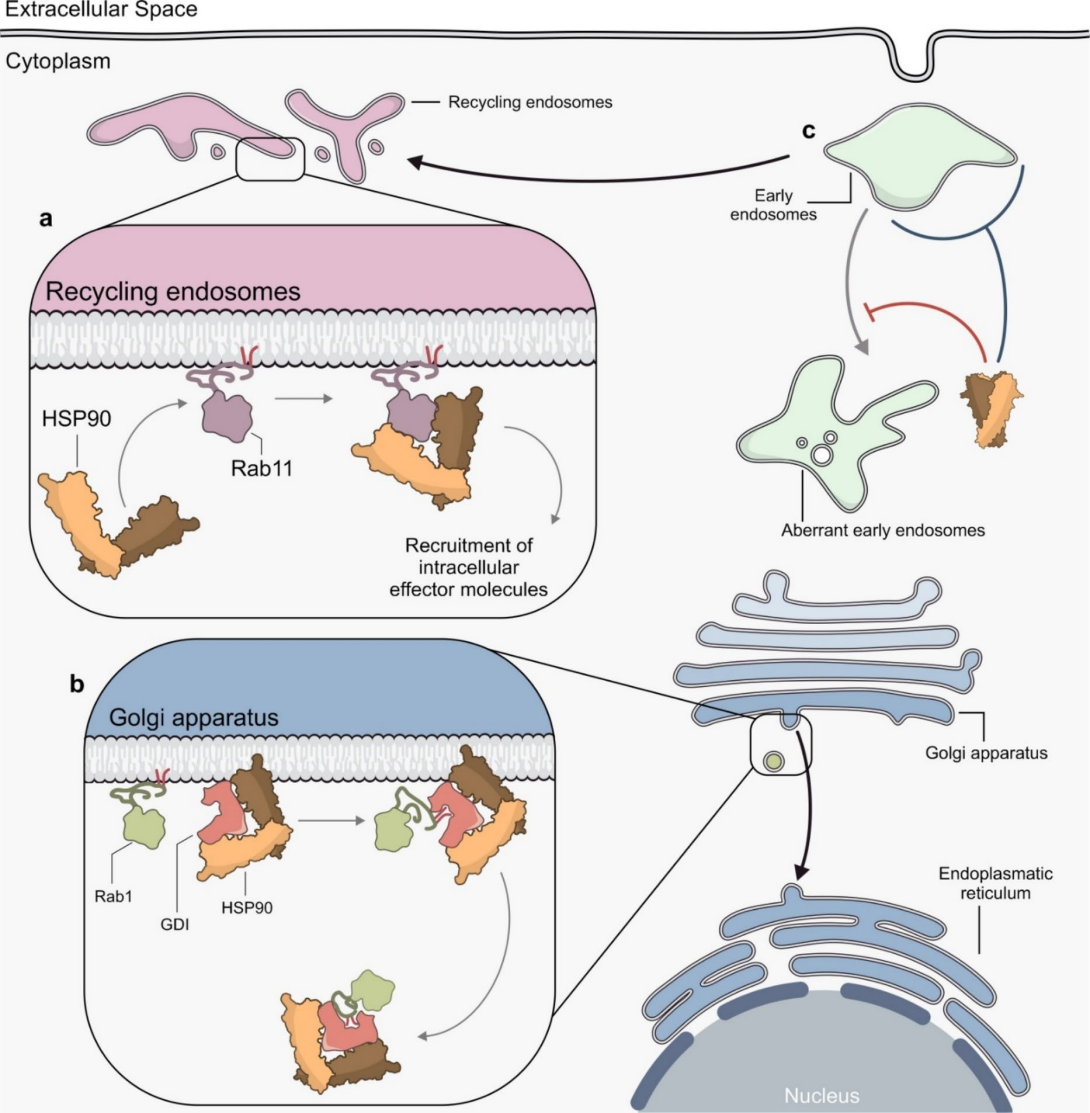
HSP90 roles in regulating Rab protein activity and endosome morphology. (**a**) HSP90 is required for promoting Rab11-positive vesicle recycling through the recruitment of intracellular effector molecules. (**b**) At the Golgi membrane, HSP90 forms a complex with Rab1 and αGDI, promoting αGDI recruitment and switch to its open conformation. This enables Rab1 to hydrolyse GTP to GDP, allowing GDI to extract Rab1 from the membrane and recycle it back to the cytosol. (**c**) HSP90 maintains the proper morphology of early endosomes and prevents the formation of tubular-like, aberrantly elongated endocytic vesicles

HSP90 has been associated with various steps of endocytosis (Table 2); one of these is the regulation of Rab protein function. Indeed, it has been found that HSP90 is involved in GDI-dependent recycling of RAB1 (Chen and Balch 2006; Raffaniello et al. 2009). RAB1 is ubiquitously expressed and is essential for ER to Golgi and intra-Golgi transport in mammalian cells (Nuoffer et al. 1994). HSP90 transiently associates with RAB1 and αGDI to form a complex localized at the Golgi membranes and this association is fundamental for the retrieval of RAB1 from the Golgi apparatus (Chen and Balch 2006). In particular, HSP90 is thought to facilitate the recruitment of the αGDI to the membrane, allowing the αGDI to switch to its open conformation. The subsequent hydrolysis of RAB-GTP to RAB-GDP allows GDI to bind to Rab thus promoting the shift of Rab geranylgeranyl lipids from the membrane bilayer to the GDI geranylgeranyl binding pocket and the dissociation of GDI-Rab complex from HSP90 into the cytosol (Chen and Balch 2006) (Fig. 5b).

**Table 2:** HSP90 functions along the endocytic pathways

HSP90 localization	Interacting Partner	Endocytic Step	Functional Outcome	Citation
Cytosolic	GDI	Golgi–ER transport	Retrieval of Rab1 from the Golgi apparatus	(Chen and Balch 2006) (Raffaniello et al. 2009)
Cytosolic	RAB11	Recycling	Endocytosis and proteolysis of c-Fms and RANK receptors	(Tran et al. 2021) (Tran et al. 2022)
Cytosolic	Not defined	Early endosomes	Control of endocytic vesicle morphology	(Cortese et al. 2013)
Cytosolic	ERBB2	Recycling	Regulation of receptor recycling	(Barr et al. 2008) (Austin et al. 2004) (Lerdrup et al. 2006) (Pedersen et al. 2008) (Cortese et al. 2013)
Cytosolic	FGF/FGFR	Recycling, degradation	Modulation of receptor translocation across endosomal membranes	(Wesche et al. 2006)
Extracellular	NadA	Recycling	Prevention of pathogen degradation in lysosomes	(Montanari et al. 2012) (Bozza et al. 2014) (Cecchini et al. 2011)
Cytosolic	Diphtheria toxin	Early endosomes	Facilitation of toxin translocation from early endosomes into the cytosol	(Ratts et al. 2003) (Schuster et al. 2017)
Membrane-bound/Cytosolic	GCRV-II viral receptor	Internalization	Mediation of viral entry via clathrin-dependent endocytosis	(Jiang et al. 2022)

Similarly, HSP90 is involved in the regulation of RAB11B activity, a RAB particularly involved in the recycling of endocytic vesicles. It was observed that HSP90 interacts with RAB11B inducing the endocytosis of some cell surface receptors in osteoclasts (OC) during osteoclastogenesis (Tran et al. 2021; Tsurukai et al. 2000). It has been recently found that RAB11B is able to induce the endocytosis of c-Fms and receptor activator of nuclear factor k B (RANK), two receptors essential for osteoclastogenesis, favouring their degradation into lysosomes (Tran et al. 2020). It was also observed that in RANK ligand (RANKL) stimulated OCs, RAB11B interacts with the ATPase domain of HSP90 and that this interaction occurs in EEs and LEs (Tran et al. 2022). Results suggested that RAB11B-HSP90 association is required for RAB11B-induced endocytosis and proteolysis of c-Fms and RANK receptors. Thus, HSP90 and RAB11B may form a complex attached to intracellular vesicles where they function in the delivery of cellular cargoes to specific cellular compartments (Tran et al. 2021) (Fig. 5a).

**Fig. 6:**
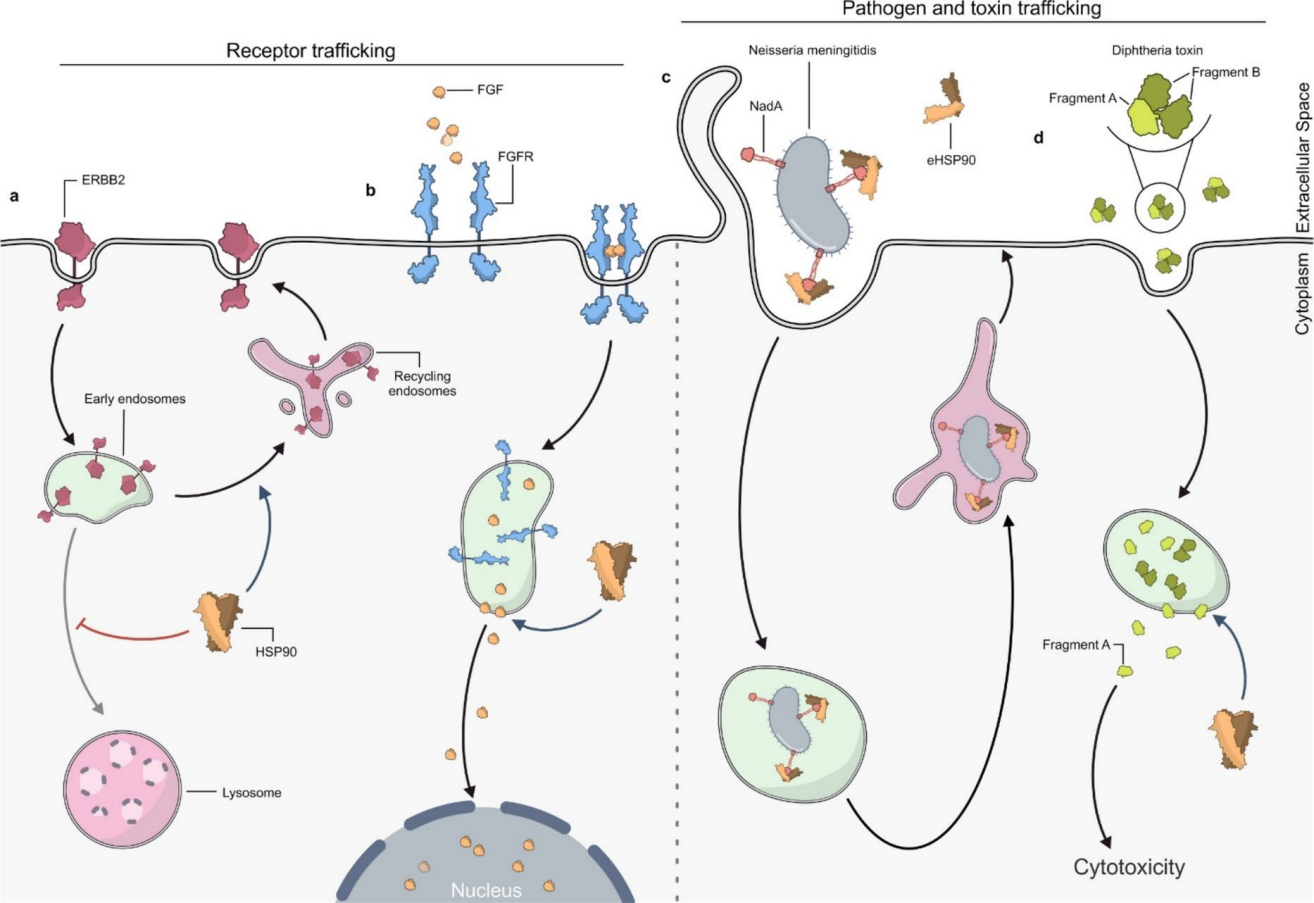
HSP90 roles in controlling surface receptors and pathogen/toxin trafficking. (**a**) HSP90 controls the endosomal trafficking of the receptor tyrosine kinase ERBB2 by modulating its endocytic route. After internalization, ERBB2 is sorted into early endosomes (EEs), where HSP90 prevents lysosomal targeting and degradation, favouring receptor recycling back to the plasma membrane. (**b**) HSP90 mediates the translocation across endosomal membranes of FGF1/2. After binding of FGF to the FGF receptor, the complex is internalized and sorted to EEs. HSP90 promotes FGF translocation across the membrane and release into the cytosol. From here, FGF translocates into the nucleus, promoting DNA synthesis. (**c**) Extracellular HSP90 (eHSP90) is involved in the endosomal trafficking of Neisseria meningitidis adhesin A (NadA). eHSP90 binds to NadA and promotes the recruitment of intracellular effector molecules that allow the pathogen to avoid lysosome targeting by entering EEs and recycling endosomes (REs), likely being transported back to the extracellular space. (**d**) HSP90 is involved in the cytosolic translocation of diphtheria toxin (DT). DT is composed of two fragments (Fragment A and Fragment B) which are cleaved on the plasma membrane and internalized. Once in EEs, HSP90 facilitates the translocation of Fragment A into the cytosol, from where it induces protein synthesis arrest and cell death

The effect of HSP90 on the endosomal trafficking of cell surface receptors has also been observed on ERBB2, a tyrosine kinase receptor associated with the onset and development of many tumour types (Austin et al. 2004; Hynes and MacDonald 2009; Marone et al. 2004). It was previously shown that the treatment of human cancerous and non-cancerous cells with the HSP90 inhibitor geldanamycin (GA) caused an increase in ERBB2 endocytosis, ubiquitination and degradation (Xu et al. 2002; Zhou et al. 2003). It has been observed that the internalization process of ERBB2 upon HSP90 inhibition occurs via either a clathrin dependent (Austin et al. 2004; Lerdrup et al. 2006; Pedersen et al. 2008) and a clathrin independent mechanism (Barr et al. 2008). It was also shown that, upon treatment with GA, ERBB2 is more localized to LEs and lysosomes rather than in recycling vesicles confirming previous results on the increase of ERBB2 degradation. It was also observed that HSP90 inhibition modified the morphology of endocytic vesicles, causing their tubular-like aberrant elongation (Cortese et al. 2013). The ultrastructure of this GA-induced compartment appears to be an intermediate between the tubular network of REs and the MVBs, likely corresponding to a transition from EEs to LEs (Fig. 5 c). Moreover, ERBB2 was observed to persistently colocalize with the transferrin receptor within elongated endosomal tubules and to be redirected together to lysosomes. Transferrin (Tf), an iron-binding glycoprotein, and its receptor (TfR) represent a classical model of the constitutive recycling pathway: after binding at the plasma membrane, the Tf-TfR complex is internalized into EEs and then rapidly recycled back to the cell surface through REs, with minimal targeting to lysosomes. This well-characterized trafficking route is commonly used as a marker to assess recycling and sorting functions. The concomitant redirection of both receptors to lysosomes suggests that these compartments may represent dysfunctional membrane domains in which recycling and sorting functions are compromised (Cortese et al. 2013) (Fig. 6a).

HSP90 seems to be fundamental also for translocation through the endosomal membrane network of other two proteins, together with their receptors, that are Fibroblast Growth Factor 1 (FGF-1) and FGF-2 (Wesche et al. 2006). FGFs bind to and activate some cell surface receptors with tyrosine kinase activity (FGFRs) (Schlessinger 2000). Once internalized, FGF/FGFR complexes enter EEs and are sorted to different subcellular compartments (Citores et al. 2001; Gleizes et al. 1996). In particular, FGF-1 and FGF-2 translocate across the endosomal membrane and are then transported into the nucleus (Klingenberg et al. 1998; Wesche et al. 2006; Wiedłocha et al. 1995; Xie et al. 2020) where they are able to induce transcription and DNA synthesis (Wiedłocha et al., 1994). Some findings indicate that radicicol, a natural antibiotic acting as a selective HSP90 inhibitor, does not interfere with the binding or internalization of FGF-1 and FGF-2, but specifically impairs their translocation across endosomal membranes (Wesche et al. 2006) (Fig. 6b).

### Involvement in pathogen entry and toxin translocation

The involvement of HSP90 in endocytosis has also been observed in the context of pathogen attacks, such as meningococcal infection, where the eHSP90 is involved. Neisseria meningitidis adhesin A (NadA) is a bacterial surface protein that allows the pathogen to get in contact with the host cell (Capecchi et al. 2005) and stimulates bacterial internalization. It was observed that a soluble recombinant NadA (rNadA) internalizes through a PI3K-dependent process mediated by the small GTPase ARF-6. Once inside the cell, rNadA avoids lysosome targeting by entering a RAB11 positive endosome recycling pathway and likely being transported back to extracellular space (Bozza et al. 2014; Takahashi et al. 2012). It was previously demonstrated that rNadA in vitro directly binds to HSP90 in its ADP-bound form (Montanari et al. 2012) and that it also binds to eHSP90 on monocyte surface (Cecchini et al. 2011). Recently, it has been proposed that eHSP90 is internalized together with rNadA in live cells and colocalizes with the recombinant protein in endocytic vesicles (Bozza et al. 2014). The use of the membrane-impermeable HSP90 inhibitor FITC-GA did not impair NadA internalization but led to intracellular accumulation of NadA positive vesicles unable to engage RAB11 and be targeted to REs. The authors speculated that rNadA-eHSP90 interaction would not serve for rNadA entry in host cells but for the recruitment of intracellular effector molecules that allow the pathogen to avoid degradation in lysosomes (Bozza et al. 2014) (Fig. 6c).

HSP90 is also implicated in the cytosolic translocation of Diphtheria toxin (DT) catalytic domain across the early endosomal membranes. DT is composed of two parts: fragment A, corresponding to the amino-terminal catalytic domain, and fragment B, encompassing carboxy-terminal and transmembrane domain(Ratts et al. 2003). DT is cleaved by a cell surface associated protease but fragment A and fragment B remain linked via an inter-chain disulphide bond that is further reduced by a reductase of the host cell (Schuster et al. 2017). The fragment B binds to the heparin-binding EGF-like growth factor that functions as a DT receptor and allows the entire DT protein to enter the cell. After internalization inside endosomes, the fragment A is released into the cytosol where it interferes with the activity of Elongation Factor 2 (EF-2) causing protein synthesis arrest and cell death (Sharma et al. 2019). The translocation of the fragment A across the EEs to the cytosol requires the presence of the cytosolic translocation factor (CTF) complex, a supramolecular complex containing HSP90. Indeed, it has been shown that HSP90 directly binds fragment A in vitro and inhibition of its chaperone activity prevents the delivery of fragment A from EEs to the cytosol both in vitro and in vivo*.* These findings suggest that HSP90 acts as a critical player facilitating cytosolic translocation of bacterial toxin (Schuster et al. 2017) (Fig. 6d).

HSP90 is also implicated in grass carp viral infection. The grass carp reovirus (GCRV) type II strain, a double-stranded RNA (dsRNA) virus belonging to the family of Reoviridae, is responsible for a high mortality rate in grass carp aquaculture. On the host cell membrane HSP90 interacts with the viral outer capsid protein VP35 and facilitates viral entry via clathrin-mediated endocytosis. Once inside the cell, HSP90 functions as a chaperone for VP35, maintaining its stability and preventing its degradation through the proteasomal pathway, thereby supporting viral replication and proliferation (Jiang et al. 2022).

Glucose-regulated protein 94 (GRP94), also named gp96 or endoplasmin, is the HSP90 isoform resident in the ER. GRP94 main activity consists of maturation and proper folding of membrane-resident and secreted protein clients and checking for misfolded molecules to dispose of them before secretion (Marzec et al. 2012). Besides its role in ER homeostasis and protein quality control, GRP94 is also involved in receptor-mediated endocytosis and, by controlling ER proteins maturation, it influences membrane client protein exposition (Pugh et al. 2022). GRP94 behaves as a low-density lipoprotein receptor-related protein 1 (LRP1, also known as CD91) ligand, stimulating LRP1-immunopeptide complex internalization, endocytosis and re-exposition in antigen-presenting cells (APCs) (Binder et al. 2000). Similar functions are played by the scavenger receptor A, which mediates GRP94 internalization in APCs. GRP94, once trafficked inside the cells, induces APC activation and antigen cross-presentation pathways that elicit immune responses (Berwin et al. 2003). In innate immune system cells, GRP94 traffics Toll-like receptors, which, in the absence of the chaperone, are not exposed to the plasma membrane, thus inhibiting immune cell microbial recognition (Yang et al. 2007; Wu et al. 2012). T and B cells require membrane exposition of α integrins to establish contacts with the bone marrow niche, an essential step for T and B cell development. B and T cells in which GRP94 expression is silenced present a defective early lymphopoiesis due to failed α integrin trafficking towards the cell membrane (Staron et al. 2010).

### HSP70

#### Isoforms, structure and functional network

**Fig. 7:**
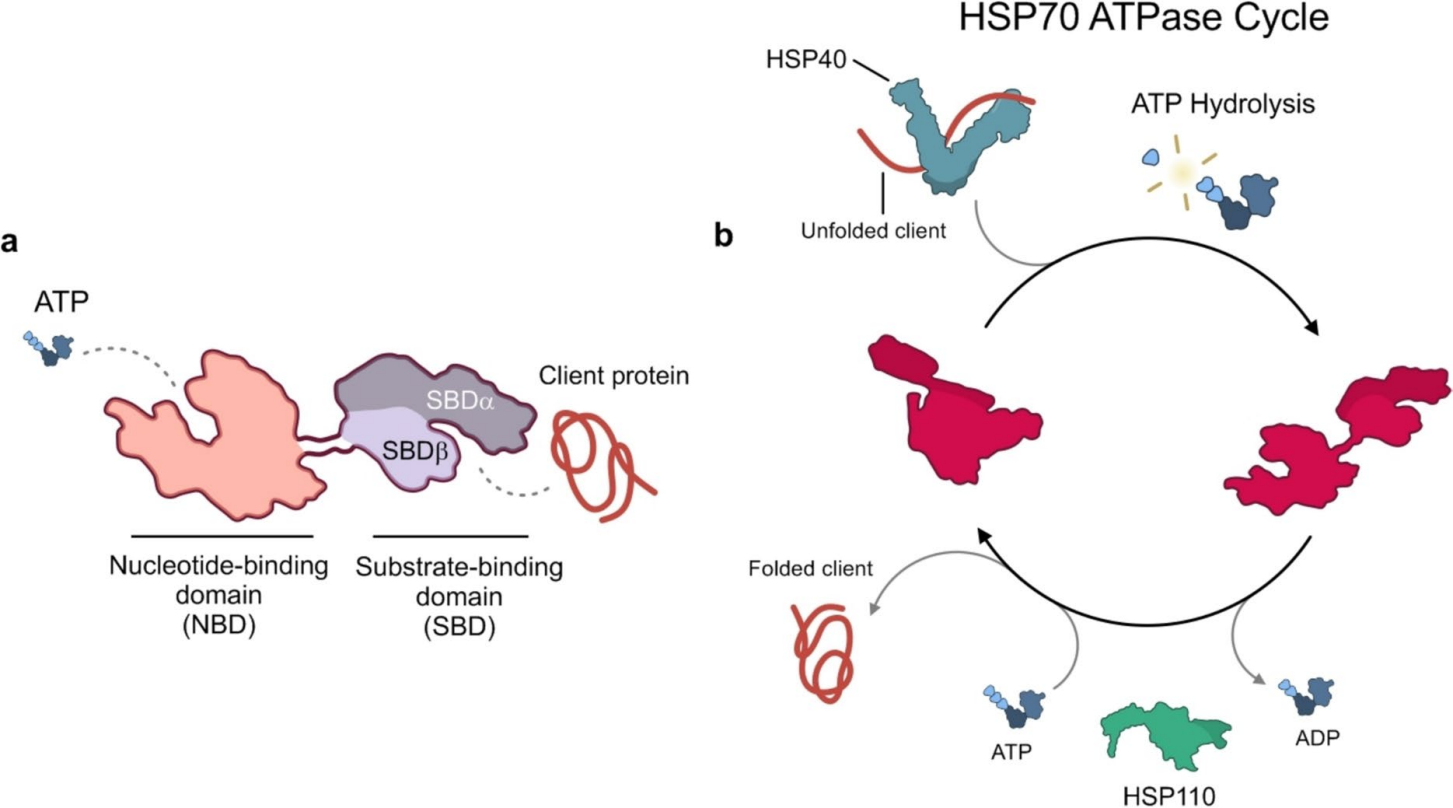
Structure, domains and ATPase cycle of HSP70. (**a**) HSP70 is composed of 2 main domains: a nucleotide-binding domain (NBD) able to bind and hydrolyse ATP, and a substrate-binding domain (SBD) which binds and folds client proteins. The SBD is also composed of two subdomains: a substrate-binding domain (SBDβ) and a helical lid domain (SBDα), in which a client peptide-binding cavity is formed. In the ADP-bound state, SBDα docks onto SBDβ to fully enclose this cavity. (**b**) HSP40 mediates the binding of substrate proteins to ATP-bound HSP70, triggering ATP hydrolysis and transition to the ADP-bound state. HSP110 then induces ADP dissociation and rebinding of ATP, converting HSP70 to the low-affinity state and consequently leading to substrate release. Subsequently, the released substrate can fold to the native state or, alternatively, re-enter the HSP70 cycle

HSP70 family comprises a variety of ubiquitous molecular chaperones that are highly conserved from bacteria to humans. In humans, 13 HSP70 homologues are expressed in distinct cellular compartments (cytosol, nucleus, ER and mitochondria) at different levels according to cellular needs (such as growth and tissue-specific activities). The human cytosolic members of these chaperones are called Heat Shock Cognate 70 (HSC70) and HSP70, with the former expressed constitutively and the latter induced upon stress and heat shock. The HSP70 chaperones possess a wide range of housekeeping functions, from folding newly synthesized proteins to disassembling protein complexes and regulating protein activity (Mayer and Bukau 2005; Rosenzweig et al. 2019). HSP70 possesses two functional domains: a nucleotide-binding domain (NBD) at the N-terminus and a substrate-binding domain (SBD) at the C-terminus, connected by a linker interdomain. The NBD binds and hydrolyses ATP to ADP to provide energy allowing the chaperone activity. The SBD is composed of a β-sheet subdomain and a α-helical subdomain, and it is responsible for binding the substrates (Rosenzweig et al. 2019) (Fig. 7a).

HSP70 works in cooperation with a network of co-chaperones such as the HSP40 protein family, also known as J-domain protein (JDP) family or DNAJ, characterized by the presence of the J-domain, and nucleotide exchange factors (NEFs), which control substrate presentation and release from HSP70s. The functional diversity of HSP70 members is further enhanced by their cooperation with other cellular chaperone systems, including sHSPs, HSP90 and HSP110, as well as protein degradation systems (Bracher and Verghese 2015; Gaur et al. 2022; Gaur et al. 2020) (Fig. 7b).

## Role in endocytic vesicular trafficking.

### Involvement in clathrin-mediated endocytosis

**Fig. 8:**
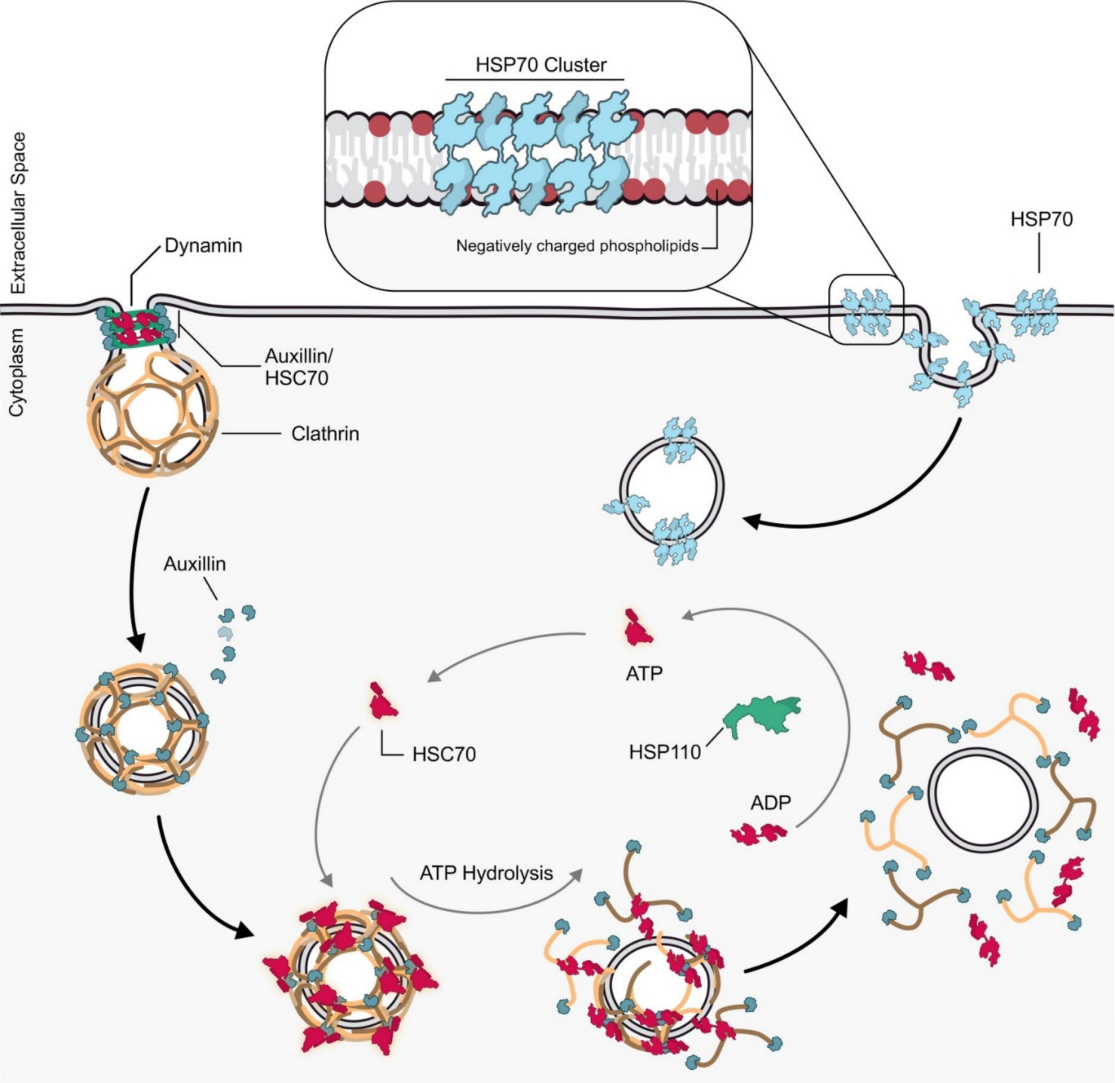
HSP70 and HSC70 roles in clathrin-mediated and clathrin-independent endocytosis. HSC70 (depicted in red), together with the J-domain-containing protein Auxilin, plays a role in the uncoating of clathrin-surrounded vesicles. HSC70 and Auxilin bind to dynamin directly; this binding helps to enrich HSC70/Auxilin at the interface between the neck and the coated pit. Clathrin vesicle scission from the plasma membrane stimulates the binding of Auxilin to clathrin. Auxilin then recruits ATP-bound HSC70 to the vesicle coat. HSC70 binds to a specific binding site at the flexible tail of the clathrin heavy chain at the vertices of the clathrin triskelion. ATP hydrolysis then exerts a mechanical force on the cage, weakening the interactions and leading to disassembly (uncoating). HSP110 binding then induces ADP-to-ATP exchange and dissociation of the complex to restart the cycle. On the other hand, HSP70 (depicted in blue) has been mainly associated with CIE. HSP70 oligomerizes and forms clusters in the plasma membrane, generating large-size nano-domains that promote endocytosis. Oligomerization is mediated by the presence of negatively charged phospholipids in the membrane

The role of the HSP70 family in the process of endocytosis has been highlighted at different steps of the pathway, from the formation of EEs to the control of recycling routes. Both HSP70 and HSC70 have been associated with CME, in particular with the uncoating of the clathrin-coated vesicles (Sousa et al. 2016; Shen et al. 2025). Indeed, once vesicle fission occurs, the clathrin cage must be rapidly removed to enable vesicle fusion with its target membrane and to recycle coat components for further rounds of vesicle budding (Pearse 1987). The uncoating of clathrin is mediated by HSC70 and cyclin-G associated kinase (GAK), a specialized clathrin-linked JDP, or its neuronal-specific homolog Auxilin (Brady et al. 2023). GAK and Auxilin share the same multi-domain structure, including the C-terminal clathrin-binding domain that allows the interaction with clathrin, but differ for the presence in GAK of an N-terminal kinase domain absent in Auxilin (He et al. 2020; Park et al. 2015). GAK is recruited to nascent clathrin-coated pits and it binds to HSC70 via its-J-domain. In this way, HSC70 can catalyze clathrin lattice remodeling from hexagons to pentagons structures, inducing membrane curvature and formation of the clathrin-coated vesicle. GAK J-domain mutants cannot recruit and activate HSC70, resulting in transient and abortive clathrin-coated pits and impaired clathrin turnover (He et al. 2025). As for GAK, the formation of the Auxilin-clathrin complex makes Auxilin able to load HSC70 close to clathrin triskelion. In this position, HSC70 hydrolyzes ATP and generates a disassembling force that drives the clathrin coat away from the vesicle. HSP110 then induces HSC70 to exchange ADP with ATP and the dissociation of HSC70 from clathrin (Rapoport et al. 2008; Sousa et al. 2016; Sousa and Lafer 2015; Xing et al. 2010). Alterations in the disassembly functions of HSC70 have pathological implications. It has been demonstrated that the aggregation of neuropathy-associated proteins leads to the sequestration of HSC70 into aberrant protein aggregates. This, in turn, reduces the levels of HSC70 available for the endocytic process, causing the reversible collapse of CME and inhibiting the internalization of membrane receptors, which affects neuronal function (Yu et al. 2014) (Fig. 8).

Auxilin, together with HSC70, is also involved in clathrin stabilization and recycling (Young et al. 2013). Indeed, Auxilin/HSC70 complex promotes the exchange of membrane-bound clathrin with cytosolic clathrin, a process that contributes to the structural flexibility of the coat and to support changes in membrane curvature during early stages of vesicle maturation (Lee et al. 2006).

Additionally, it was found that both HSC70 and Auxilin bind to dynamin directly and independently of one another, suggesting that dynamin may serve to enrich HSC70/Auxilin at the interface between the neck and the coated pit, promoting clathrin turnover and facilitating the constriction and fission of the invaginated pit. Auxilin inhibits the GTPase activity of dynamin, preventing premature or excessive activity, ensuring that dynamin only constricts the vesicles at the right moment. It has been demonstrated that Auxilin-dynamin interaction is important for clathrin-coated vesicle formation (Newmyer et al. 2003). In newly forming pits, GAK allows the recruitment of clathrin and of the adaptor proteins AP1 and AP2, which connect clathrin to selected cargoes (McMahon and Mills 2004). This likely depends on the ability of GAK to phosphorylate the mu1 and mu2 chains of AP1 and AP2 (Lee et al. 2005).

It was also observed that cells subjected to heat shock display an increase in transferrin CME, that is blocked by inhibiting HSP70 expression by siRNAs (Vega et al. 2010), suggesting that HSP70 plays a role in transferrin endocytosis. In proximal tubular kidney cells (PTCs), nucleus-specific HSP70 isoform HSPA1L plays a similar function, protecting cells from diabetic kidney disease (DKD). It forms a complex with the adipokine vaspin and the clathrin heavy chain, mediating vaspin internalization and intracellular trafficking, preserving organelle homeostasis under DKD and obesity-related stress conditions (Nakatsuka et al. 2021).

HSC70 has also been implicated in CME of certain viruses. Notably, in these cases, HSC70 is not only involved in the uncoating of clathrin-coated vesicles but it also plays a role at the cell surface, where it can act as a receptor for viral entry. For example, in transmissible gastroenteritis virus (TGEV), the viral M protein interacts with surface-localized HSC70 (sHSC70). Knockout (KO) of HSC70 leads to the accumulation of viral particles at the cell membrane, indicating that sHSC70 is essential for virus entry. Furthermore, HSC70 ATPase activity is required for the uncoating of viral containing vesicles (Chen et al. 2024; Ji et al. 2023).

Similarly, surface HSC70 has been shown to participate in rotavirus entry (Wang et al. 2020). Rotavirus invasion of epithelial cells is known to involve multiple sequential interactions with different cell surface receptors (Amimo et al. 2021). HSC70 appears to mediate a later stage of this entry process (Guerrero et al. 2002). Notably, treatment of cells with monoclonal antibodies against HSC70 significantly inhibits rotavirus infection, suggesting that HSC70 is required for successful viral entry into host cells (Gutiérrez et al. 2010).

### Involvement in clathrin-independent endocytosis

Surface HSP70 (sHSP70) is also involved in CIE. Interestingly, it was observed that sHSP70 oligomerizes and forms clusters on cell surface, generating large sized nano-domains that promote endocytosis. Oligomerization is thought to be mediated by the linker inter-domain and by the C-terminal region of the SBD. Interfering with these oligomerisation interfaces, by using recombinant HSP70 variants that lacks these regions, was shown to impair endocytosis (Nimmervoll et al. 2015) (Fig. 8).

In specific cases, HSP70 and HSC70 seem to display opposite roles in cargo trafficking. Indeed, it has been found that the two proteins differentially traffic the epithelial sodium channel (ENaC) in oocytes. Analysis of HSC70 and HSP70 functions in Xenopus oocytes demonstrated their antagonistic relationship, with HSC70 decreasing the surface exposition and functionality of the ion channel and HSP70 enhancing ENaC functional expression and trafficking to the membrane (Goldfarb et al. 2006).

### Association with membranes

It could be speculated that the different mechanisms through which HSP70 and HSC70 in some cases operate may be related to the distinct capacity of these proteins to interact with membranes (Vega et al. 2010). Both proteins can directly associate with lipid bilayers strongly interacting with negatively charged phospholipids, such as palmitoyl-oleoyl-phosphoserine (POPS) and cardiolipin (CL). However, they display different affinities for specific phospholipids. In particular, HSP70 and HSC70 differ in their association with palmitoyl-oleoyl-phosphocholine (POPC): HSC70 maintains a partial affinity, whereas HSP70 does not interact with POPC at all (Arispe and De Maio 2000; Vega et al. 2008). Moreover, both proteins can induce in vitro liposome aggregation, although with distinct properties in the assembly process (Armijo et al. 2014; Dores-Silva et al. 2021). At low HSP concentration, the rate of liposome aggregation is similar for both proteins, but at higher levels, HSC70 and HSP70 behave differently. HSP70 binds to liposomes more rapidly, whereas HSC70 interacts with the lipids more slowly but triggers a stronger aggregation. These dynamics suggest different affinity of the two HSP70s for lipidic membranes (Arispe et al. 2002).

Besides cytosolic members, other HSP70 homologues have also been shown to interact with lipid membranes and have been implicated in endocytic or membrane-associated processes (Dores-Silva et al. 2020a; Dores-Silva et al. 2020b). GRP78, also known as HSPA5 or BiP, is the ER-resident form of HSP70. Despite being an ER-resident chaperone, it was shown to translocate to the plasma membrane where it associates with negatively charged phospholipids and inserts into lipid bilayers through the synergistic action of its N- and C-terminal domains (Dores-Silva et al. 2020a). In colon and lung cancer cells, ER stress induces PERK-Akt-mTOR signalling activation, which results in GRP78 loading into ER-derived vesicles thank to the action of the co-chaperone DNAJC3. ER-derived vesicles firstly fuse with EEs and then with REs, which finally mediate the exposition of GRP78 and other ER-resident chaperones to the cell surface, where they interact with surface partners and induce cancer cell survival and metastasis (Gonzalez-Gronow et al. 2009; Van Krieken et al. 2021). Once exposed at the cell surface, GRP78 has also been reported to function as a receptor or co-receptor facilitating viral entry (Chu et al. 2018; Ibrahim et al. 2020; Zhou et al. 2023). The mitochondrial form of HSP70, also known as HSPA9, mtHSP70 or mortalin, has been reported to interact with membranes, particularly with bilayers -enriched in CL, a phospholipid abundant in the inner mitochondrial membrane (IMM). In vitro studies showed that HSPA9 inserts very efficiently into CL-containing liposomes, whereas it displays reduced affinity for membranes with lower CL content (Dores-Silva et al. 2020b).

### HSP40

The DNAJ/HSP40 family consists of HSP70 co-chaperones characterized by the presence of an evolutionarily conserved J-domain, which is essential for stimulating the ATPase activity of HSP70. HSP40 binds to non-native proteins, presenting them to HSP70 and thereby determining client specificity and fate (Qiu et al. 2006; Jiang et al. 2019). Members of the HSP40 family can also function independently of HSP70 and, by acting synergistically with each other, control protein aggregation. HSP40s have well-established roles in various pathologies, including cancer and neurodegenerative diseases (Qiu et al. 2006; Hasegawa et al. 2017). While some HSP40 family members, such as Auxilin/GAK, are known to interact with and cooperate with HSC70/HSP70 to regulate clathrin-mediated endocytosis, other HSP40 proteins have distinct and independent roles in endocytic processes. One of these chaperones is Cysteine String Protein α (CSPα, also known as DNAJC5), a chaperone protein that is generally located to synaptic vesicles and secretory granules. CSPα directly interacts with dynamin and facilitates its polymerization during vesicle fission, even if the molecular mechanism is unknown. In addition to endocytosis defects in CSPα KO cells, it has been also observed an impairment in the recycling vesicle pool suggesting that CSPα is also involved in the recycling trafficking (Sheng and Wu 2012).

CSPα has also been described to regulate EGFR endocytosis. In lung adenocarcinoma (LUAD), CSPα expression is higher than in adjacent normal tissues and it correlates with poor prognosis in LUAD patients. Proteomic analyses identified a direct interaction between CSPα and EGFR, with the intracellular domain of EGFR required for stabilizing this binding. Upon EGFR stimulation, CSPα overexpression promotes EGFR internalization into EEs and its downstream signalling. Mechanistically, CSPα associates with both EGFR and the clathrin adaptor protein AP2A1, strengthening their interaction and thereby facilitating EGFR recruitment into clathrin-coated pits and promoting endocytosis (Chen et al. 2025). CSPα has been also identified as a key component of the host cell machinery required for proper intracellular trafficking of the bacterial toxin ExoU (Deruelle et al. 2021), a potent cytotoxin delivered by the Pseudomonas aeruginosa into the host cell (Chamberlain et al. 2022; Juan et al. 2017). Once inside the cytosol, ExoU colocalizes with CSPα-positive vesicles, which transport the toxin from the perinuclear region toward the cell periphery. Functional studies demonstrated that mutations in CSPα do not prevent ExoU from binding to intracellular membranes but impair its trafficking inside the cell, suggesting that the chaperone is specifically required only for vesicular transport rather than for initial membrane association. Initial screening analysis of this infection process identified only CSPα as a host factor involved, highlighting the potential value of this chaperone as a therapeutic target. (Deruelle et al. 2021).

Another member of the HSP40 chaperone family, that can work independently of HSP70, is the mammalian homolog of Receptor Mediated Endocytosis 8 (RME-8, gene DNAJC13), a ubiquitously expressed J-domain-containing protein. RME-8 localizes mainly to endosomal membranes and it is involved in endocytosis and intracellular trafficking dynamics (Xhabija and Vacratsis 2015). Particularly, it has been found to play an important role in the endocytosis of the EGFR. EGFR can enter cells via clathrin-mediated mechanisms and via caveolae, and the activation of these two pathways depends on the concentration of EGF (Sigismund et al. 2018; Sapmaz and Erson-Bensan 2023). RME-8 knockdown did not affect the caveolae-mediated endocytic pathway suggesting that the chaperone only influences clathrin-mediated endocytosis. Although RME-8 does not colocalize with clathrin-coated structures at the plasma membrane, RME-8 knockdown has been observed to alter the distribution of clathrin-heavy chains (CHCs). Under normal conditions, CHCs are found throughout the cytosol, often appearing as puncta near the plasma membrane where clathrin-coated vesicles form. After RME-8 knockdown, clathrin distribution is altered and about 30% of cells shows reduced cytosolic staining (Girard et al. 2005). 

## Small chaperones in the endocytic mechanisms

**Fig. 9:**
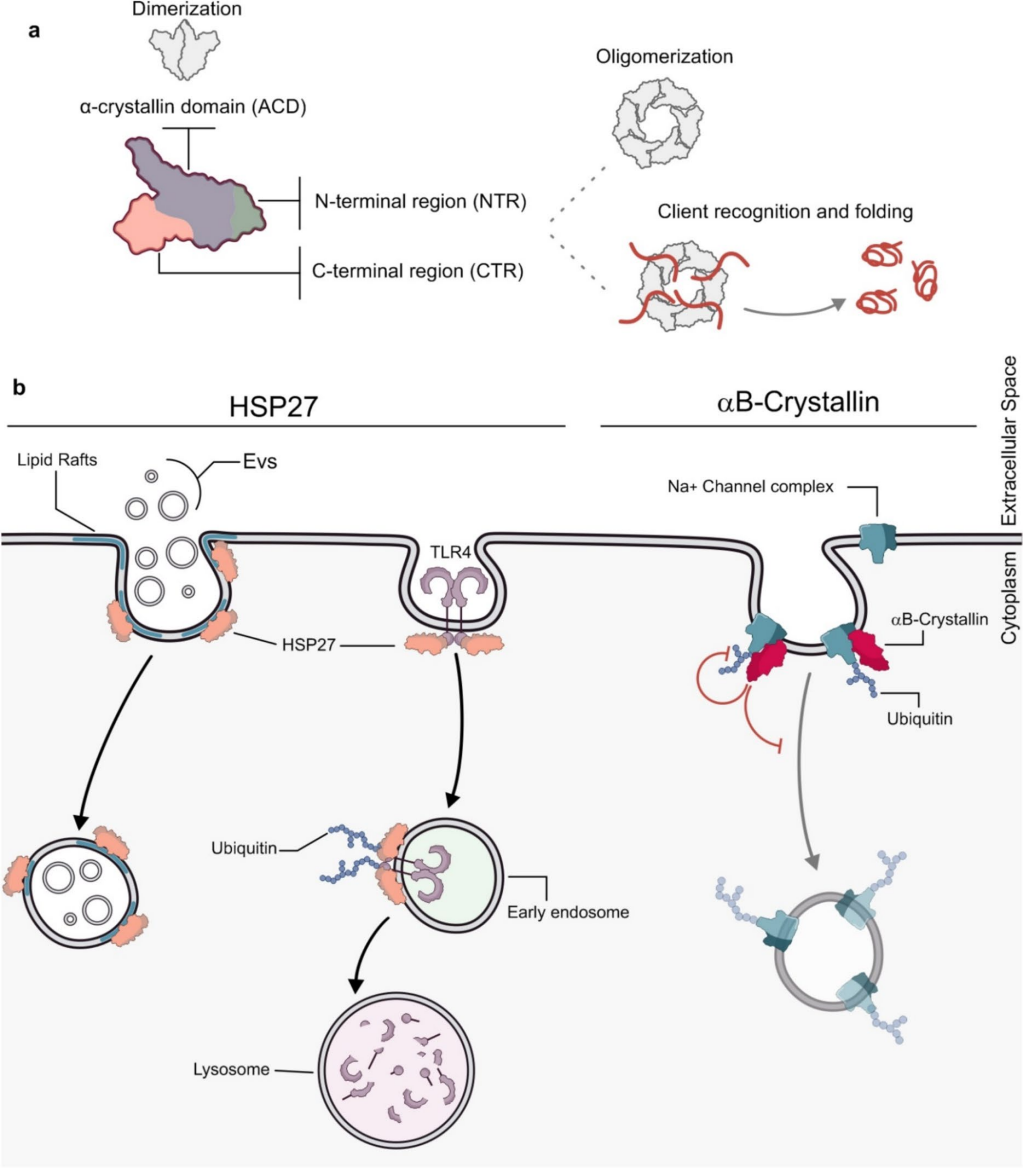
Selective roles of small chaperones in endocytic pathways. (**a**) Small HSPs are characterized by the presence of three domains: a highly conserved α-crystallin domain which mediates dimerization, creating the building blocks of higher oligomers. The α-crystallin domain is flanked by an N-terminal domain (NTD) and a C-terminal domain (CTD). NTD and CTD can substantially vary among sHSPs but are crucial for oligomerization and client recognition and folding (Gu et al. 2025). (**b**) HSP27 triggers exosome uptake through lipid-raft-mediated endocytosis; it also promotes ubiquitination and subsequent degradation of TLR4 by directly interacting with it. αB-crystallin modulates the endocytosis of the cardiac voltage-gated Na + channel complex by inhibiting its ubiquitination, thereby stabilizing the channel at the plasma membrane

Isoforms, structure and subcellular localizationsHSPsare chaperone proteins with low molecular weight, highly conserved throughout the evolution in all the kingdoms (Carra et al. 2017). They are structurally composed of a non-conserved N-terminal region (NTR) of variable length, a conserved α-crystallin domain (ACD) and a non-conserved short C-terminal region (CTR) (Janowska et al. 2019; Reinle et al. 2022). sHSPs function independently of ATP and they do not display refolding activities. sHSPs can form homo and heterodimers or oligomers, reaching a molecular weight of around 900 kDa (Mymrikov et al. 2020) (Fig. 9a). The sHSP family comprises ten members, from HSPB1 to HSPB10: most of them (B1, B2, B5, B6, B7 and B8) are ubiquitously expressed, while the others show a tissue-specific expression. HSPB3 (also known as HSPL27) is mainly present in the heart, skeletal and smooth muscles and nervous system (La Padula et al. 2016; Sugiyama et al. 2000), while HSPB4 (also known as αA-Crystallin) is highly expressed in eye lenses (Woods et al. 2023). HSPB9 (also known as CT51) and HSPB10 (also known as ODF1) are testis-specific (Tedesco et al. 2022); HSPB9 expression is confined in testis germ cells and varies during spermatogenesis (de Wit et al. 2004), while HSPB10 has also been observed in kidney ducts (Cabrillana et al. 2019; Tedesco et al. 2022).

Morgana and Melusin are also included among the small chaperones (Johnson 2021). Besides possessing an α-crystallin domain (Garcia-Ranea et al. 2002), they carry two cysteine and histidine-rich domains (CHORD) at the N-terminal. Morgana is ubiquitously expressed (Poggio et al. 2024; Ferretti et al. 2010), while Melusin is exclusively expressed in striated muscle tissues and its expression is not upregulated by heat stress, but by an increase in mechanical stretch (Sbroggiò et al. 2008; Sorge and Brancaccio 2016; Acquarone et al. 2025). Both Melusin and Morgana can operate as HSP90 co-chaperones or function independently (Ferretti et al. 2011; Michowski et al. 2010; Sorge et al. 2024).

### Role in endocytic vesicular trafficking

It is known that HSPs indirectly modulate vesicle trafficking and membrane dynamics by interacting with cytoskeletal elements, which are important players in endocytosis (Lavoie et al. 1993; Wettstein et al. 2012; Chen et al. 2023). In addition, evidence has shown that HSP27 (also known as HSPB1) and αB-Crystallin (HSPB5), can directly associate with lipid membranes displaying different binding profiles. At higher concentration, HSP27 reaches saturation upon incorporation into liposomes, particularly those enriched in POPS. In contrast, αB-Crystallin shows a linear incorporation pattern within the same concentration range (De Maio et al. 2019). The α-crystallin domain, a key feature of these proteins, seems to be crucial for membrane insertion since, after incorporation of the two sHSPs into POPS liposomes, this domain was found to be protected from proteinase K digestion (De Maio et al. 2019). These membrane-association properties may provide the molecular basis for the functional involvement of sHSPs in endocytic processes. Indeed, some indications are emerging on specific roles of sHSPs in regulating endocytic pathways. Recently, HSP27 has been shown to play a role in exosome uptake via lipid-raft-mediated endocytosis in glioblastoma cells (GBM). Short-term incubation of GBM cells with exosomes leads to a 2- to 4.5-fold increase in phosphorylation of several lipid-raft-associated proteins, such as the extracellular signal-regulated kinase (ERK) and HSP27. The inhibition of ERK1/2 signalling resulted in a dose-dependent decrease in HSP27 phosphorylation levels, suggesting that HSP27 was phosphorylated downstream of ERK1/2 during exosome uptake. Furthermore, a direct role for HSP27 in exosome internalization was confirmed by a significant reduction in exosome uptake following siRNA-mediated knockdown of HSP27. These findings indicated that exosome lipid-raft mediated endocytosis depends on the activation of the ERK1/2 signalling pathway, with HSP27 playing a role as an effector molecule (Svensson et al. 2013) (Fig. 9b). HSP27 has also been observed to play a role in the endocytosis and degradation of Toll-like receptor 4 (TLR4) following lipopolysaccharide (LPS) stimulation in THP1 monocytic cells (Li et al. 2019). TLR4 is a key receptor in the innate immune response, particularly in the recognition of bacterial infections. Its main ligand, LPS, is a major component of the outer membrane of Gram-negative and some Gram-positive bacteria (Vaure and Liu 2014). It has been shown that phosphorylated HSP27 (p-HSP27) can bind to TLR4, inhibiting its signalling activity and promoting its internalization. Particularly, it has been observed that p-HSP27 disrupts the interaction between TLR4 and MD-2, a soluble protein that bind to the extracellular domain of TLR4 and is required for LPS-induced inflammation. Once internalized, TLR4 accumulates in Rab5-positive vesicles, where HSP27 promotes receptor ubiquitination and trafficking toward lysosome degradation (Bucci et al. 1992; Li et al. 2019) (Fig. 9b).

Recently, the chaperonin containing TCP-1 subunit 4 (CCT4), a component of the cytosolic chaperonin CCT, has been identified as a small chaperone relevant for vesicle trafficking and protein secretion. CCT4 regulates protein secretion in both *C. Elegans* and mammalian cells by stabilizing the Golgi and recycling vesicle structure, and it promotes vesicle trafficking towards the plasma membrane by folding tubulin and dynamin (Chen et al. 2023).

αB-Crystallin also plays a role in endocytosis and vesicle trafficking regulation. Specifically, it regulates the cell surface expression and internalization dynamics of Na_v_1.5, the pore-forming α subunit of the primary cardiac voltage-gated Na^+^ channel complex. αB-Crystallin directly interacts with Na_v_1.5 and its overexpression stabilizes Na_v_1.5 on cell surface by decreasing its ubiquitination. On the other hand, the knockdown of αB-Crystallin led to a marked reduction in the surface expression of Na_v_1.5. Previous studies have indicated that the ubiquitination of Na_v_1.5 is mediated by the ubiquitin ligase neural precursor cell expressed developmentally downregulated gene 4-like (also known NEDD4-2) and results in a decrease of cell surface Na_v_1.5 levels. Co-immunoprecipitation analysis revealed that αB-Crystallin interacts with NEDD4-2 and that this binding affects the interaction between NEDD4-2 and Na_v_1.5. Overall, αB-Crystallin inhibits Na_v_1.5 ubiquitination, endocytosis and degradation by preventing its association with its ubiquitin ligase NEDD4-2 (Huang et al. 2016).

## Discussion

**Fig. 10:**
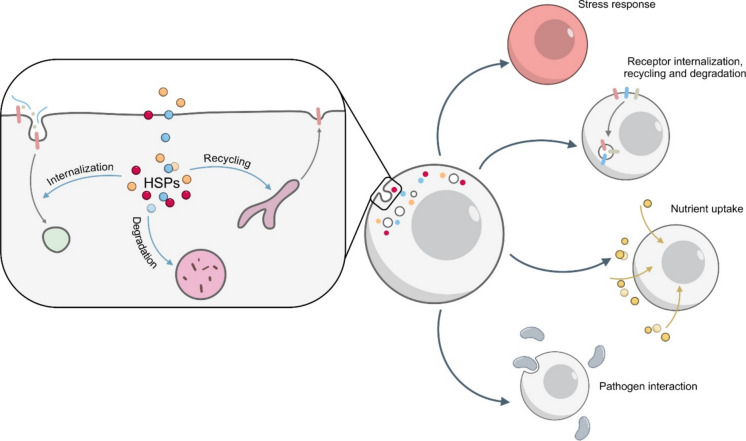
Summary of heat shock protein functions in controlling endocytosis. Heat shock proteins (HSPs) control key steps of the endocytic pathway, including receptor internalization, cargo recycling, and degradation. By modulating these processes, HSPs influence stress responses, receptor turnover and signalling, nutrient uptake, and cellular interactions with pathogens

While protein folding is a well-known function of HSPs, their involvement in endocytosis is still only partially characterized. Several studies have reported their involvement in different steps of molecule uptake, vesicle trafficking, and cargo sorting, but the overall picture of how HSPs contribute to the endocytic network is still incomplete (Fig. 10).

A central question is whether HSPs act as independent players or function in a coordinated way. Indeed, some evidence indicates that they can display both cooperative and antagonistic effects. For example, HSP70 promotes transferrin receptor internalization and recycling, thereby enhancing iron uptake, whereas HSP27 and HSP90 seem to counteract this activity (Vega et al. 2010; Chen et al. 2006). In another instance, HSP90 facilitates antigen escape from endosomes into the cytosol, thus favouring cross-presentation, while HSP70 can direct antigens toward lysosomal degradation and MHC II presentation (Kato et al. 2012). In the context of viral infections, HSP70 and HSP90 seem to act in cooperation, with HSP90 mediating the earliest stage of viral entry and HSP70 contributing to downstream steps (Lubkowska et al. 2021).

A crucial feature is the ability of HSPs to directly interact with cellular membranes regulating vesicle formation, cargo recruitment and Rab protein activation at specific compartments (Dores-Silva et al. 2020a; Van Krieken et al. 2021; Honda et al. 2009; Dores-Silva et al. 2020b; Chu et al. 2018). Emerging data suggest that distinct HSPs display selective affinities for membranes with different lipid compositions, which may underlie the functional specialization of family members such as HSP70 and HSC70 (Arispe et al. 2002). Understanding these preferences could shed light on how HSPs discriminate among vesicular subpopulations and tailor their activities to distinct trafficking routes.

Additionally, HSPs such as HSP70 and HSP90 are well known to facilitate protein translocation across membranes (Hartl 1991; Craig 2018; Cyr and Neupert 1996; Wesche et al. 2006). These established roles highlight the ability of chaperones to interface with membranes, a property that may also underlie their emerging functions in endocytosis and vesicle trafficking.

Under stress conditions, HSP availability and function may be drastically reprogrammed, impacting on their housekeeping roles. Initially, stress can divert HSP activity away from constitutive processes like endocytosis, toward emergency activities, such as preventing protein aggregation. However, when the HSR is triggered, the expression of many HSPs is strongly upregulated, allowing cells not only to cope with emergency but also to restore and even boost HSP housekeeping functions (Alagar Boopathy et al. 2022). In this sense, endocytosis has been proposed to be a stress-regulated process. For instance, transferrin internalization is enhanced after heat shock (Vega et al. 2010; López-Hernández et al. 2020) and exposure to heat stress can enhance phagocytic activity in immune cells (Vega and De Maio 2005). An increase in endocytosis in response to stress may contribute to cell survival for several reasons: (1) the internalization and removal of damaged membrane components and surface receptors is important to preserve a functional interface with the extracellular environment (Vega et al. 2010; López-Hernández et al. 2020); (2) receptor internalization through endocytosis contribute to sustain signalling pathways activated under stress conditions (López-Hernández et al. 2020); (3) stressed cells require increased nutrient uptake to generate energy and to replace damaged proteins and membrane components (Vega et al. 2010); (4) cell migration, another stress-induced process, is also supported by endocytosis. Indeed, efficient migration requires the internalization and coordinated recycling of integrins, transmembrane receptors that interact with the extracellular matrix enabling cell motility (Lang et al. 2012; Nikolaou and Machesky 2020; Paul et al. 2015).

Overall, central questions regarding many aspects of HSP role in regulating endocytosis remain open. First, it is unclear whether HSPs operate independently or perhaps forming stable molecular complexes and dynamic molecular machineries able to modulate the endocytic processes. Equally important is understanding how stress conditions impact HSP-mediated regulation of endocytosis, whether this effect is cell- or tissue-specific and how it varies depending on the nature and intensity of the stress.

Addressing these issues will require innovative experimental approaches. Single-cell and omics-based technologies would capture the heterogeneous complexity of HSP functions in endocytosis, while the use of primary cells and 3D models would clarify physiological relevance. Additionally, novel in vitro methods, such as lipid-overlay assays, lipid pull-down assays and liposome microarray-based assays (LiMA) (Saliba et al. 2015), could be employed to investigate protein–lipid interactions. Protein–protein interaction analyses, including co-immunoprecipitation, mass spectrometry, and proximity labelling proteomics approaches, such as such as BioID (proximity-dependent biotin identification) (Roux et al. 2012) and APEX (engineered ascorbate peroxidase proximity labelling) (Nguyen et al. 2020) would help to map dynamic chaperone complexes. Moreover, advanced imaging techniques, including super-resolution microscopy and cryo-EM, would provide nanoscale visualization and structural insights into HSP-client complexes, supporting a deeper mechanistic understanding of these processes. Finally, CRISPR/Cas or RNAi-mediated modifications, used in addition to pharmacological inhibitors, could further strengthen the results obtained so far.

In summary, HSPs emerge as versatile regulators of endocytosis whose activities are dynamically tuned by stress, membrane composition and cooperative interactions with other chaperones. Understanding how these factors integrate will be crucial to uncover the full contribution of HSPs to vesicular trafficking under both normal and pathological conditions.

## Data Availability

Not applicable.
